# Quality and resource efficiency in hospital service provision: A geoadditive stochastic frontier analysis of stroke quality of care in Germany

**DOI:** 10.1371/journal.pone.0203017

**Published:** 2018-09-06

**Authors:** Christoph Pross, Christoph Strumann, Alexander Geissler, Helmut Herwartz, Nadja Klein

**Affiliations:** 1 Department of Healthcare Management, Berlin University of Technology, Straße des 17. Juni 135, 10623 Berlin, Germany; 2 Institute for Entrepreneurship and Business Development, University of Lübeck, Ratzeburger Allee 160, 23562 Lübeck, Germany; 3 Chair of Econometrics, Georg-August-University Göttingen, Humboldtallee 3, 37073 Göttingen, Germany; 4 Melbourne Business School, University of Melbourne, 200 Leicester Street, Carlton VIC 3053, Australia; Florida International University, UNITED STATES

## Abstract

We specify a Bayesian, geoadditive Stochastic Frontier Analysis (SFA) model to assess hospital performance along the dimensions of resources and quality of stroke care in German hospitals. With 1,100 annual observations and data from 2006 to 2013 and risk-adjusted patient volume as output, we introduce a production function that captures quality, resource inputs, hospital inefficiency determinants and spatial patterns of inefficiencies. With high relevance for hospital management and health system regulators, we identify performance improvement mechanisms by considering marginal effects for the average hospital. Specialization and certification can substantially reduce mortality. Regional and hospital-level concentration can improve quality and resource efficiency. Finally, our results demonstrate a trade-off between quality improvement and resource reduction and substantial regional variation in efficiency.

## 1 Introduction

Most OECD countries are characterized by a steady increase of hospital expenditures, which account for a substantial and increasing share of overall health care costs [1–3]. Reforms have been implemented to induce cost containment in the hospital sector, e.g., the introduction of activity-based hospital budgets. Increasing cost pressures have triggered concerns that hospitals face a trade-off between quantity of patients treated and quality of care provided [4–7]. As a consequence, the importance of hospital quality in managerial decision-making, policy-making, and health economics research is increasing. Hospitals have to optimize the relationship between cost and quality in order to provide high quality services using as few resources as possible. In particular, hospital clinical and administrative managers today often have to decide where to reduce resource investments while ensuring continued high levels of service quality [8]. Furthermore, for hospitals with different environmental characteristics and for different medical conditions, the optimal balance between service quality and efficiency might differ substantially [9].

Both cost and quality of health care have been shown to vary widely among countries, regions, and providers [10–14], suggesting an enormous potential for quality improvements and cost reductions. Regarding stroke care, for example, 30-day mortality increases by more than 8 times when moving from the best emergency care units to the worst emergency care units in the Berlin metropolitan area. At a national level, this difference is even more dramatic [15]. Despite these differences the importance of outcome service quality of medical services has not been fully reflected in the hospital efficiency literature [16]. Due to the lack of appropriate measures to incorporate quality in efficiency analysis, and difficulties in hospital quality measurement itself, models of hospital efficiency have rather focused on ad-hoc output measures such as patient and procedure volumes. The most important hospital production component, quality of care, has often been neglected [17–19].

In a seminal paper, [20] apply Stochastic Frontier Analysis (SFA) to estimate a multi-product cost function. To account for outcome quality differences and distinct patient populations, they include quality metrics as output variables, including a case-mix index (CMI) and 30-day risk-adjusted mortality. Their results emphasize the sensitivity of efficiency estimates with regard to the inclusion of quality measures. [21] highlight the non-trivial impact of including outcome quality measures or patient burdens of illness on efficiency estimates and hospital rankings. Based on cross-sectional regressions, [22] find higher cost inefficiency associated with a higher mortality rate in Florida hospitals, but in a separate study they fail to detect an association between cost inefficiency and care outcomes [23]. The authors explain these contradictory results with regional differences in the relationship between hospital quality and cost inefficiency. In summary, most approaches to include quality into hospital efficiency estimations are restricted to stylized Data Envelopment Analysis (DEA) models and obtain inconclusive results [18, 24–28]. As a consequence, little of the hospital efficiency research has been translated into policy or service delivery [29, 30].

The existing studies are often limited to cross-sectional data, hospital samples are restricted regionally or to a single ownership group. Quality indicators are often not risk-adjusted, and rarely include extended time-frames after hospital discharge. Likewise, hospital efficiency and outcome quality is often examined for the entire hospital or jointly for several medical conditions, which is problematic, especially when outcome quality is considered, given condition-specific risk and resource profiles [31]. Furthermore, the existing hospital efficiency literature has rarely accounted for spatial patterns of hospital performance, which have been detected, e.g., for Germany [32, 33] or for England [34, 35]. Neglecting common performance patterns of hospitals in the same or neighboring regions might induce systematic biases to inefficiency scores and estimated effects of their determinants, if quality varies not only between providers but across regions [36–38]. Stressing the importance of taking into account geographic clusters, [39] analyze the effectiveness of the US Hospital Readmission Reduction Programm (HRRP) in reducing hospital readmission and find that localizing benchmarks to reduce dispersion of readmission rates makes hospital quality improvement initiative more effective.

As demonstrated, existing studies on hospital efficiency face important limitations that impact their validity and generalizability, and have limited the relevance for policy and management applications. However, developing a more robust and comprehensive method to estimate hospital efficiency including quality of care is important to aid medical, policy and managerial decision makers on allocation of limited resources and the difficult balance between resource efficiency and quality of care improvement in health care systems that face demands for expanded services and increasing cost pressures.

To respond to this challenge, this study, proposes and implements a new methodology to estimate quality-adjusted hospital efficiency at scale. We further apply this model to a specific medical condition to investigate the determinants of hospital performance. Our aim is to identify the effects of care specialization, service line certification and treatment concentration on the efficiency of care provision for a hospital’s risk-adjusted patient pool. As production output, we propose a risk-adjusted and medical condition-specific measure of hospital patient volume. This modification reduces the risk that unmeasured differences in hospital outputs affect estimated efficiencies. In contrast to [20], we do not further adjust the output term by quality, but rather include realized outcome quality as inputs. Holding the output term constant, hospitals are encouraged to minimize poor quality (mortality and readmissions) and resources in order to treat patients efficiently. Thus, we propose a more comprehensive notion of (risk-adjusted) hospital performance that includes both quality of care and resource efficiency. Moreover, technical efficiency is captured at the medical condition and medical department as the relevant levels of analysis [40], which differentiates between quality-adjusted technical efficiency of different medical departments even within one hospital. At the same time, we take correlated spatial patterns among hospitals into account.

We focus our analysis on the acute care for stroke patients, which is characterized by high variation of quality of care [41, 42]. Quality of care in stroke treatment is especially relevant for survival, disability, and long term cost of care. Stroke is a leading cause of disability, morbidity, and mortality in both developed and developing countries, with 6.7 million deaths globally each year [43]. Due to demographic changes and high costs of treatment, follow-up, and rehabilitation, stroke care poses a major challenge to the entire health care system. At the same time, advances in stroke care promise improvements in care outcome quality [44–46], and associated reductions in long term cost of care [47]. Hospitals adopt these care improvements to different degrees creating quality performance differentials between care providers.

For the empirical implementation, we merge an information rich unbalanced panel data set for the time period 2006 to 2013 with a newly proposed, Bayesian geoadditive SFA model. The data set includes structural and quality metrics at the hospital and medical department level, with a focus on standardized stroke mortality (SMR) and readmissions, as well as the structural determinants of stroke outcomes, such as stroke unit capacities and certification. Recently proposed spatial SFA models control for spatial dependence by specifying either spatial lags of the output variable [48], of the input variables [49] or of the inefficiency term [50]. However, none of these models do control for individual heterogeneity, which is taken into account by other non-spatial SFA models [51–53]. The flexibility of the applied Bayesian SFA approach exceeds the flexibility of the former models by allowing a distinguished analysis of individual heterogeneity and observable characteristics of inefficiency, while accounting for space dependent patterns of local performance by including geoadditive predictors of technical inefficiencies. Based on estimated efficiency scores we determine slack resources and estimate the saving potential in terms of quality improvements (saved deaths and readmissions) and resource reallocations if stroke care is provided efficiently. We thus aim to develop a methodology that can help multi-hospital provider groups and health service regulators to assess quality-adjusted technical efficiency to identify best-practice providers and identify potential for quality and efficiency improvements.

## 2 Data and methods

### 2.1 Data

The empirical approach is based on an unbalanced panel data set, which combines four distinct data sources and comprises annual observations for 2006 until 2013 for more than 1,100 stroke treating German acute care hospitals. The panel data set is unique in time coverage, number of hospitals included, and scope of variables at the medical department, hospital and regional level. The panel data set provides a novel opportunity to jointly evaluate hospital efficiency and quality of care for a specific medical condition and at the medical department level.

As a first data source, we process structural hospital and medical department data (e.g., ownership, number of beds, disease and procedural case volumes) from publicly accessible hospital report cards, which are available for 2006, 2008, 2010, 2012 and 2013. The report cards are part of the mandatory external quality monitoring system initiated in the early 2000s and operated by the executive authority in the German health care system, the Federal Joint Committee (Gemeinsamer Bundesausschuss, G-BA). Resource inputs (physician and nurse staff levels) are aggregated from the relevant medical departments and weighted by the share of stroke patients treated in each department. This provides a more exact measurement of the resource inputs at the relevant unit of analysis.

As a second data source, we integrate stroke outcome quality, such as the risk-adjusted 30-day mortality ratio and 30-day readmissions, as well as stroke case volumes, computed by the hospital report card initiative Quality Assurance with Routine Data (QSR). The QSR scheme is operated by the largest German sickness fund the AOK (Allgemeine Ortskrankenkasse), and employs administrative in- and outpatient data of AOK insured patients. The outcome quality measures cover three different stroke types: *Intracerebral hemorrhage* (ICD Code I61, 12% of all cases in Germany in 2014), *Ischemic stroke* (I63, 86%) and *Stroke not specified as hemorrhage or ischemic stroke* (I64, 2%). The AOK uses its administrative patient data to calculate risk-adjusted outcome rates for each hospital. The data enables detailed annual risk-adjustments by means of logistic regressions that include patient-specific risk-factors such as age, gender, and co-morbidities (diabetes, hypertension, etc.) [54]. The indicators cover a 30-day time period after hospital discharge, which is important for a more comprehensive quality of care assessment.

As a third data source, we integrate stroke unit information from the German Stroke Society (Deutsche Schlaganfall Gesellschaft, DSG). The data covers, inter alia, information on which hospitals operate DSG-certified stroke units and the time period of certification. The DSG stroke unit certificate stipulates an integrated, co-located, and high resource stroke care model that takes into account evidence-based care guidelines [55], which in many dimensions go beyond the average (non-certified) stroke unit infrastructure. As documented in Table 1, for 2013 the data set includes 219 hospitals with DSG-certified stroke units (with on average 619 stroke patients), 235 hospitals with a non DSG-certified stroke unit (340 stroke patients), and 937 hospitals without a stroke unit (64 stroke patients).

**Table 1:** Means and sums of selected variables (2013).

	all	cert. SU	not cert. SU	no SU	others
number of hospitals	1883	219	235	937	492
*hospital size*					
beds	270.6	658.5	435.0	226.0	104.4
inpatient cases (in k)	10.1	25.9	17.5	8.7	2.2
*stroke patients*					
sum of stroke patients (in k)	274.5	135.5	79.9	60.0	-
share of stroke patients (in %)	100	49.4	29.1	21.8	-
ICD diagnoses^a^ per hospital	145.8	618.9	339.9	64.0	-
OPS procedures^b^ per hospital	119.0	679.8	322.2	0.2	-
QSR case volume per hospital	70.7	204.9	117.1	22.7	-
*QSR quality indicators*^c^					
standardized mortality ratio^d^	1.0	0.9	0.9	1.0	-
observed mortality rate (in %)	14.8	12.3	12.8	16.0	-
observed readmission rate (in %)	13.6	11.8	12.2	14.3	-

^*a*^Includes ICD codes I61, I63 and I64.

^*b*^OPS procedure volume can be higher than ICD diagnoses volume because OPS procedures are also applied in case of other ICD diagnoses not included in this article´s set of ICD stroke diagnoses (e.g., G45 = transient ischemic attack).

^*c*^Simple average across hospitals without weigthening based on patient volume.

^*d*^The risk-adjusted mortality ratio relates the observed stroke mortality to the expected stroke mortality based on the number of patients treated and their risk-profiles.

Lastly, we integrate regional district-level data on number of general practice physicians and economic indicators from the INKAR database hosted by the German Federal Institute for Research on Building, Urban Affairs, and Spatial Development.

### 2.2 The Bayesian geoadditive stochastic frontier model

In comparison with DEA analyses, flexible SFA models have an important advantage. SFA addresses the concerns that DEA models quantify all departures from the best practice frontier as inefficiencies, including those caused by random events and measurement errors. This might lead to an overestimation of inefficiency [56]. For the modelling of hospital efficiency including quality of care metrics, random events and measurement errors are of particular concern.

In a stylized form, SFA models of technical efficiency consist of a formalized production technique linking an output and input variables, and a random deviation from the production technique that comprises idiosyncratic noise and technical inefficiencies. We rely on a flexible SFA model proposed by [57] that builds upon earlier contributions, such as [58], [59], [60], [61], [51], [52], [53]. While SFA has become a standard parametric approach in modelling efficiency and efficiency-enhancing potentials in the provision of goods or services by economic entities such as firms, households, farms, and hospitals, the incorporation of spatial dependence in SFA models and other deterministic approaches to inefficiency modelling is in its infancy. The model addresses the important distinction between (time-invariant) determinants of inefficiency from patterns of unobserved heterogeneity by means of scaling of random inefficiency [52, 53]. Formally, the model reads as

$$\begin{array}{ccc} y_{it} & = & \eta_{it}^{\left(y\right)}+v_{it}-u_{it},\\ & = & \eta_{it}^{\left(y\right)}+v_{it}-u_{it}^{*}\exp(\eta_{it}^{\left(u\right)}), \end{array}$$ (1)

where *y*_*it*_ denotes the (log) output of individual (i.e., hospital) *i* at time *t*, $\eta_{it}^{\left(y\right)}$ and $\eta_{it}^{\left(u\right)}$ are predictors shaping the deterministic component of production and the distribution of technical inefficiencies, respectively. Concrete specifications of these predictors can be found in Eqs (6) and (7). The term *v*_*it*_ is an idiosyncratic noise term and $u_{it}^{*}$ is a positive random variable invoking a wedge between actual output and its efficient counterpart. Joint with the formalization in (1), the following assumptions constitute the observation model that we use to evaluate quality of health care provision:

1. The predictor $\eta_{it}^{\left(y\right)}$ allows to relate the observable output *y*_*it*_ to covariates, and individual and time effects. Metric covariates may contribute linearly to $\eta_{it}^{\left(y\right)}$ or in a more complex nonlinear manner which is formalized by means of so-called penalised splines [62, 63]. In addition, the predictor might process information inherent in categorical variables. The composition of the Bayesian regression design is specific for all these effect types. We refer to [57] for a more detailed discussion of model representations for geoadditive SFA regression, as well as further methodological references therein to the broad framework of (Bayesian) structured additive regression.
2. To describe random deviations of actual output from the efficient frontier, technical inefficiency *u*_*it*_ obeys the following structure
  $$\begin{array}{c} u_{it}=u_{it}^{*}\alpha_{it},\;\alpha_{it}=\exp(\eta_{it}^{\left(u\right)}),\;u_{it}^{*}∼N^{+}(\mu_{u}^{*},{\left(\sigma_{u}^{*}\right)}^{2}), \end{array}$$ (2)
  where $N^{+}$ is short for the truncated normal distribution nesting the half normal distribution $\left(\mu_{u}^{*}=0\right)$ as a special case. At the empirical side, small means μ* > 0 might be difficult to detect in light of the composite disturbance that describes deviations between actual and efficient output. Scaling technical inefficiency as in (2) has been first proposed by [52]. As an implication, *u*_*it*_ exhibits a truncated normal distribution with parameters
  $$\begin{array}{c} \mu_{it}=\mu_{u}^{*}\alpha_{it}\;\text{and}\;{\left(\sigma_{u}^{2}\right)}_{it}={\left(\sigma_{u}^{*}\right)}^{2}\alpha_{it}. \end{array}$$ (3)
3. Economic entities providing goods or services are subject to measurable and unobservable local conditions. Accordingly, the predictor $\eta_{it}^{\left(u\right)}$ allows a decomposition as
  $$\begin{array}{c} \eta_{it}^{\left(u\right)}=\eta_{it}^{\left(o\right)}+f_{spat}\left(dist_{i}\right), \end{array}$$ (4)
  where $\eta_{it}^{\left(o\right)}$ relates inefficiency to observable covariates, and *f*_*spat*_(*dist*_*i*_) accounts for unobservable spatial conditions of (in)efficient output provision of the district hosting the *i*-th entity (denoted *dist*_*i*_). As formalized in (4), the ‘district’ might be considered to provide a third data dimension apart from the individual hospital and time dimension. [64] provide strong evidence for the informational content of random regional effects for the modelling of hospital efficiency in Germany. The rightmost component in (4) allows a further distinction into spatially (i.e., positively) connected effects (*f*_*struct*_(*dist*_*i*_)) and purely random effects (*f*_*unstruct*_(*dist*_*i*_)), i.e.,
  $$\begin{array}{c} f_{spat}\left(dist_{i}\right)=f_{struct}\left(dist_{i}\right)+f_{unstruct}\left(dist_{i}\right). \end{array}$$ (5)
  The structured part (*f*_*struct*_) refers to a specific neighbourhood structure being a (symmetric but possibly weighted) relation based on, e.g., common borders, cross-border care models, and cross-regional academic medical centers. In a Bayesian formulation, such effects can be modelled by means of Gaussian Markov random field priors [65]. Similar to temporal autoregressions, the systematic part of spatial dependence shrinks with the distance between districts so that the positive correlation among neighbouring districts captured by means of *f*_*struct*_(*dist*_*i*_) generalizes similar structures as so-called spatially autoregressive models [66]. Altogether, the complete effect *f*_*spat*_ in (5) subjects economic performance to geographical information, which likely invokes performance dependence among neighbouring districts, but also allows the occurrence of pure random effects to capture district-specific differences.
4. As an implication of the nonlinear model structure that results from the scaling of $u_{it}^{*}$, the predictor structure for $\eta_{it}^{\left(y\right)}$ and $\eta_{it}^{\left(u\right)}$ may overlap. For identification purposes $\eta_{it}^{\left(u\right)}$ does not contain an intercept, as this is represented by the scale of $u_{it}^{*}$.
5. The idiosyncratic noise *v*_*it*_ and innovations to inefficiency $u_{it}^{*}$ are independent within and across all entities and time.

In its most flexible specification, the SFA approach in [57] allows the use of predictors for all distribution parameters and hence also for *σ*_*v*_, the specification of $\mu_{u}^{*}\neq 0$, nonlinear effects for metric variables or different prior structures for all involved unknown parameters. In this analysis, we focus on *f*_*spat*_ offering a geoadditive resolution of quality of health care and add the following assumptions to facilitate model identification and interpretation: (i) The predictors for $\sigma_{u}^{*}$, *σ*_*v*_ contain solely intercepts while $\mu_{u}^{*}$ is set to zero, and (ii) metric covariates are modelled linearly to capture effects of such variables by a single slope coefficient. On the implementation side, we (i) assume flat priors for all coefficients of linear effects, while (ii) for the unit and district-specific random effects we specify multivariate Gaussian priors with zero mean vectors and marginal variances that themselves are supposed to have inverse gamma prior distributions. To capture the structured spatial effect by Markov random field priors, (iii) the districts are considered as neighbours if they share common borders. The strength of the spatial structure is controlled by a variance parameter with inverse gamma hyperprior.

### 2.3 Stochastic frontier modelling of quality and efficiency

The formal representation in Eq (1) is operationalized in terms of an input-oriented stroke quality production function. For the output, we utilize the volume of stroke patients adjusted for their risk-profiles. It is computed by scaling-up the expected 30-day stroke mortality for hospital *i* at time *t*, *ExpMor*_*it*_, (calculated by the QSR initiative based on patient risk profiles and patient volumes [54]) with the inverse of the hospital average annual 30-day observed mortality rate at time *t*, $\bar{ObsMorRate_{t}}$, i.e. $RisAdjPatVol_{it}=ExpMor_{it}/\bar{ObsMorRate_{t}}$.

We specify two distinct types of input variables. First, the 30-day observed mortality (*ObsMor*_*it*_) and the number of readmitted patients after 30 days (*ObsReadm*_*it*_) serve as measures of quality of care. Second, to account for resource use, we include the number of (full-time equivalent) physicians (*Phys*_*it*_) and nurses (*Nurs*_*it*_) as input variables.

Relying on a Cobb-Douglas production function, we follow [67] to correct for zero input values, i.e., the optimal level of realized quality—no deaths or readmissions—by including two dummy variables ($D_{it}^{ObsMor}$) and ($D_{it}^{ObsReadm}$) in the production function. The variables take on the value of 1 when observed mortality or readmission are zero, respectively. This procedure allows us to preserve a substantial proportion of sample observations with an optimal level of quality, i.e., zero values for one or both of the variables *ObsMor*_*it*_ and *ObsReadm*_*it*_. Neglecting these observations might result in seriously biased estimators of the parameters of the production function [67]. The empirical production function reads as

$$\begin{array}{ccc} \log(RisAdjPatVol_{it}) & = & \beta_{0}+\beta_{1}D_{it}^{ObsMor}+\beta_{2}D_{it}^{ObsReadm}\\ & & +\beta_{3}\log\left(ObsMor_{it}^{*}\right)+\beta_{4}\log\left(ObsReadm_{it}^{*}\right)\\ & & +\beta_{5}\log\left(Phys_{it}\right)+\beta_{6}\log\left(Nurs_{it}\right)\\ & & +\lambda_{t}+v_{it}-u_{it}^{*}\exp\{\eta_{it}^{\left(u\right)}\}, \end{array}$$ (6)

where $D_{it}^{k}=1$ if *k*_*it*_ = 0, $D_{it}^{k}=0$ if *k*_*it*_ > 0 and $k_{it}^{*}=\max\left(k_{it},D_{it}^{k}\right)$ with *k* ∈ {*ObsMor*, *ObsReadm*}. Year effects are included as λ_*t*_ for *t* = {2008, 2010, 2012, 2013}, with 2006 providing the reference year. We specify the inefficiency term ($\eta_{it}^{\left(u\right)}$) in (6) as

$$\begin{array}{ccc} \eta_{it}^{\left(u\right)} & = & f_{spat}\left(dist_{i}\right)+\tau_{t}+\delta_{1}Spec_{it}\\ & & +\delta_{2}MSStrDis_{it}+\delta_{3}NumStrHospDis_{it}+\delta_{4}\log\left(PatShaStroke_{it}\right)\\ & & +\delta_{5}MedDepCon_{it}+\delta_{6}SUCert_{it}+\delta_{7}SUnonCert_{it}+\delta_{8}GPsPerDis_{it}\\ & & +\delta_{9}\log\left(HosBed_{it}\right)+\delta_{10}PrivHos_{it}+\delta_{11}NonProfHos_{it}+\delta_{12}Teach_{it}\\ & & +\delta_{13}UniHos_{it}+\delta_{14}ShaI61_{it}+\delta_{15}ShaI64_{it}+\delta_{16}DiagCon_{it}, \end{array}$$ (7)

where the variables governing hospital inefficiency ($\eta_{it}^{\left(u\right)}$) allow a classification into six groups: specialization, certification, centralization, outpatient care, spatial structure and other control variables. Similar to the production function we also model year effects for the inefficiencies with *τ*_*t*_.

#### Stroke specialization

We include a hospital-level stroke specialization measure in form of the stroke patient share of all inpatient cases treated annually in a hospital (*PatShaStroke*_*it*_). With a relatively high stroke patient share, stroke treatment process and investment requirements have increased priority for the hospital and its staff. Likewise, staff is more experienced in and focused on stroke treatment. In previous studies, medical focus through specialization has been shown to have a positive impact on hospital quality performance, both through positive spillover effects and commonalities [68].

More importantly, we consider the existence of a stroke unit as a specific structural measure of specialization. In stroke units, care is provided by an interdisciplinary team of experts, including neurologists, cardiologists, radiologists and neuro- and vascular surgeons. These experts are co-located in a specialized site with 24/7 availability and dedicated stroke diagnostic equipment such as CT scanners. These infrastructure and procedural conditions allow accurate and early diagnosis of type and extend of stroke. The [44] found consistent evidence that more organized stroke care is associated with improved outcome quality, especially in seperate stroke units with dedicated staff and facilities. We capture stroke unit specialization through the variable *SUnonCert*_*it*_, which marks hospitals that perform 10 or more complex stroke procedures (German procedure (OPS) codes 8-891 and 8-89b) in a stroke unit annually [69], but have not received a special stroke service line certification.

#### Certification

Hospital certificates confirm the compliance with general structural, process, and quality management standards or specific treatment guidelines set by medical specialty associations. To get approved, hospitals have to reach those higher-than-normal standards and fix systematic problems, which is supposed to result in better and more efficient inpatient care. However, empirical evaluations of the benefits of certification have shown inconclusive results with regard to improvements of patient safety and quality of care [70–73]. In our empirical model, the stroke unit variable *CertSU*_*it*_ differentiates hospitals that have received a stroke unit certification from the German Stroke Society (DSG) from other hospitals.

#### Centralization

Centralization of service provision has been demonstrated to improve outcome quality and reduce costs [74–77]. In particular, centralization can lead to a more optimal regional care model based on economic considerations, patient needs, and quality of care. However, political resistance often hinder centralization of care [78]. In this study, we distinguish inter- and intra-hospital centralization. The former is quantified at the district level via hospital market shares for stroke patients (*MSStrDis*_*it*_), as well as the number of hospitals in one district treating stroke patients (*NumStrHospDis*_*it*_). The latter is included in form of a Herfindahl Hirschman Index (HHI) of the medical department stroke patient shares within one hospital (*MedDepCon*_*it*_).

#### Outpatient care

General practitioners (GPs) and specialists in outpatient care monitor stroke risk factors, such as obesity, high blood pressure, and diabetes. GPs also provide the continuum of care after hospital discharge, in particular post-hospital observation and recovery from in-hospital conditions. GP density and continutiy of care can thus positively affect inpatient outcome quality if outpatient care is readily available in the hospital district [79–82].

#### Underlying spatial structures

Several studies find that quality of care varies across regions [36, 37]. Moreover, as shown by [33], spatial clusters characterize German hospital performance, and [35] find similar effects for England. Ignoring this form of dependence can affect estimation accuracy and might also induce systematic biases to inefficiency scores and estimated effects of their determinants [66]. Moreover, if spatial clusters exist for both the dependent and explanatory variables, estimated relationships might appear stronger than they actually are [38]. Due to the difficult definition of a hospital market, we consider two rival spatial structures to ensure robustness of the empirical findings. On the one hand, we rely on the districts to define a hospitals’ region and, on the other hand, on a broader classification based on the European Nomenclature of Units for Territorial Statistics (NUTS2) level. However, these artificial market boundaries cannot be assumed to reflect the true hospital markets, which might have an overlapping structure, e.g., due to patient flows. To capture both, correlated (overlapping) structures between the regions and region-specific effects, we separate the geoadditive effects in a structured part assigning a Markov random field prior, and an unstructured effect with identically distributed Gaussian prior (see also Section 2.2 for details). Moreover, through both effects, we are able to account for regional differences in health status and behaviour.

#### Control variables

The empirical model also includes control variables common in the empirical hospital literature, such as hospital size measured through the number of hospital beds (*HosBed*_*it*_), ownership type (*PrivHos*_*it*_ and *NonProfHos*_*it*_), university hospital (*UniHos*_*it*_), teaching status (*Teach*_*it*_) and general, hospital-level specialization (*Spec*_*it*_). While other studies include hospital beds as production inputs, we treat beds as an exogenous efficiency influence for two reasons. First, in Germany the number of hospital beds for each medical speciality is fixed through state-level hospital plans in the short- and mid-term [83]. Second, the total number of hospital beds, which aggregates number of beds in the different medical departments, does not characterize information pertinent to the actual medical departments that treat stroke patients. We control for overall hospital-level specialization, as specialized hospitals and medical departments can respond more rapidly and comprehensively to unanticipated and rare treatment complications. Specifically, we employ the [84] specialization measure, which captures specialization by treatment area depending on volume thresholds, i.e., the average number of patients treated nationally (or 80% of the hospital’s patients) in a specific diagnostic category (*Spec*_*it*_). Furthermore, the particualar stroke types are associated with different resource requirements and distinctive survival and complication risks [85, 86]. To account for this, we include the composition of stroke patients for each hospital, which is approximated by the share of hemorrhage stroke relative to ischemic stroke (*sh* − *I*61_*it*_ and *sh* − *I*64_*it*_), as well as the distribution of the 3 types (*DiagCon*_*it*_). Lastly, we control for the number of thrombectomies conducted in each hospital (*ShaTRHOMB*_*it*_) to take into account high resource requirements, better treatment outcomes and more severe stroke cases associated with thrombectomy treatments. Table 2 shows some descriptive statistics of the considered variables.

**Table 2:** Descriptive statistics: Mean and standard deviation (in parentheses).

Variable	2006	2008	2010	2012	2013
number of hospitals	1133	1121	1085	1096	1087
*output variable*					
log(*RisAdjPatVol*)	3.74 (1.17)	3.62 (1.31)	3.39 (1.47)	3.28 (1.52)	3.28 (1.59)
*input variables*					
*D*^*ObsMor*^	0.08 (0.27)	0.11 (0.31)	0.15 (0.36)	0.15 (0.36)	0.16 (0.36)
*D*^*ObsReadm*^	0.09 (0.29)	0.11 (0.32)	0.16 (0.36)	0.18 (0.38)	0.17 (0.37)
log(*ObsMor* *)	1.80 (1.11)	1.74 (1.17)	1.62 (1.25)	1.56 (1.27)	1.52 (1.29)
log(*ObsReadm* *)	1.55 (1.07)	1.53 (1.17)	1.47 (1.23)	1.44 (1.24)	1.46 (1.25)
log(*Phys*)	-0.58 (1.39)	-0.60 (1.56)	-0.67 (1.69)	-0.65 (1.71)	-0.62 (1.77)
log(*Nurse*)	0.64 (1.39)	0.54 (1.55)	0.50 (1.73)	0.49 (1.77)	0.53 (1.82)
*explanatory variables*					
*Spec*	1.37 (0.75)	1.34 (0.74)	1.46 (0.83)	1.36 (0.81)	1.35 (0.81)
*MSStrDis*	0.31 (0.31)	0.31 (0.32)	0.31 (0.33)	0.29 (0.32)	0.30 (0.33)
*NumStrHospDis*	6.69 (8.13)	6.93 (8.52)	6.85 (8.57)	7.52 (9.15)	7.48 (9.21)
log(*PatShaStroke*)	-4.42 (1.16)	-4.50 (1.30)	-4.61 (1.44)	-4.62 (1.53)	-4.66 (1.51)
log(*HosBed*)	5.52 (0.77)	5.51 (0.78)	5.51 (0.80)	5.50 (0.81)	5.51 (0.81)
*PrivHos*	0.17 (0.38)	0.18 (0.38)	0.19 (0.39)	0.21 (0.41)	0.21 (0.41)
*NonProfHos*	0.44 (0.50)	0.44 (0.50)	0.45 (0.50)	0.43 (0.50)	0.43 (0.50)
*Teach*	0.39 (0.49)	0.43 (0.49)	0.47 (0.50)	0.50 (0.50)	0.52 (0.50)
*UniHos*	0.03 (0.18)	0.03 (0.18)	0.03 (0.18)	0.03 (0.18)	0.03 (0.18)
*MedDepCon*	0.87 (0.19)	0.87 (0.19)	0.85 (0.19)	0.84 (0.20)	0.83 (0.20)
*ShaI*61	0.09 (0.10)	0.10 (0.10)	0.11 (0.11)	0.11 (0.11)	0.11 (0.10)
*ShaI*64	0.18 (0.22)	0.11 (0.18)	0.10 (0.18)	0.09 (0.18)	0.09 (0.17)
*DiagCon*	0.69 (0.18)	0.73 (0.17)	0.73 (0.17)	0.73 (0.16)	0.73 (0.16)
*SUCert*	0.08 (0.26)	0.07 (0.25)	0.11 (0.31)	0.10 (0.30)	0.11 (0.32)
*SUnonCert*	0.14 (0.35)	0.20 (0.40)	0.25 (0.43)	0.24 (0.42)	0.24 (0.43)
log(*GPsPerDis*)	3.93 (0.16)	3.90 (0.16)	3.88 (0.16)	3.86 (0.16)	3.84 (0.17)

*refers to $k_{it}^{*}=\max\left(k_{it},D_{it}^{k}\right)$, where $D_{it}^{k}=1$ if *k*_*it*_ = 0 and $D_{it}^{k}=0$ if *k*_*it*_ > 0 with *k* ∈ {*ObsMor*, *ObsReadm*}

## 3 Results

In this section, we present our results in five steps. First, in order to base the empirical analysis on the appropriate model, we evaluate various model specifications, which differ with regard to the inclusion of unobservable heterogeneity. Second, we display parameters describing the variation in the quality-expanded notion of hospital performance. The findings are checked for robustness against alternative performance definitions by excluding some input variables. Third, we analyze spatial patterns of inefficiencies in care provision when controlling for unobservable local conditions of hospital efficiency. Fourth, based on estimated efficiency scores and respective marginal effects of the underlying explanatory variables, we quantify slack resources and the associated potential for quality improvements or resource reductions as efficiency improvements. Lastly, we subject our results to a series of robustness checks.

In all models, we employ a total of 120,000 Markov Chain Monte Carlo (MCMC) iterations. To reduce autocorrelations, we delete the first 20,000 iterations (burn-in) and store each 100th iterate (thinning). We check convergence of the chains graphically in terms of the sampling paths and autocorrelation plots. To ensure numerical stability, we center the output and input variables by subtracting their means. For the discussion of empirical results, we regard particular effects to be ‘significant’ if a certain posterior credibility interval does not contain zero effects. The models were estimated in the open source software BayesX [87], where an implementation of the geoadditive SFA models is provided.

### 3.1 Model selection

The diagnostic results of alternative models are displayed in Table 3. Starting with hospital individual effects, we extend the model by random effects varying on a regional level to take unobservable local conditions into account. Germany comprises 438 districts or 38 NUTS2 regions for which we allow spatial dependence patterns of technical inefficiencies. While each NUTS2 region hosts at least one hospital, the sample comprises hospitals which are located in 415 of the 438 districts. In consequence, for the specified spatial effects, the random Markov field (structured spatial pattern) obtains effects for all districts, while unstructured (random) spatial contributions are only determined for districts that host at least one hospital.

**Table 3:** Model selection criteria for distinct specifications of spatial structures.

		districts			NUTS2		
	no spatial	struct & unstruct	struct	unstruct	struct & unstruct	struct	unstruct
	(1)	(2)	(3)	(4)	(5)	(6)	(7)
*DIC*	2817.6	**2581.7**	2591.0	2595.7	2766.7	2768.0	2766.2
*WAIC*	2975.8	**2742.3**	2750.8	2750.9	2933.2	2935.2	2932.3

based on 5522 observations (i.e. 1294 hospitals in 415 district and 38 NUTS2 regions)

To assess model fit, we use the deviance information criterion (DIC) [88]. The DIC of the model neglecting any spatial dependence (Model 1) exceeds the DIC of rival models. The specification of region-specific random effects to explain variations in inefficiency improves model accuracy substantially. This result indicates the presence of regional patterns in hospital performance. In general, the district level (Models 2–4) is more appropriate to account for these unobservable local conditions than the broader NUTS2 level (Models 5–7). The specification of both, a structural and a non-structural spatial random effect at the district level, achieves the best model fit (i.e., the lowest DIC). This is also confirmed in terms of the Watanabe-Akaike information criterion (WAIC) [89]. As a consequence, the following discussion of the results is mainly based on Model 2.

### 3.2 Estimated parameters

Table 4 provides estimated parameters of the best fitting quality-and-resource Model 2 along with the results for two models with alternative input specifications concentrating each only on one performance dimension, the quality-only Model 8 or the resource-only Model 9. On the left hand side of Table 4, we show the estimated coefficients for the production function and the model fit results for the three different models. On the right hand side, we show the estimated coefficient for the inefficiency terms. In order to analyze potential trade-offs between outcome quality and staff resources, we include the input variables as explanatory variables describing variations of inefficiencies when they are not incorporated in the production function. We also display effects (standardized coefficients) resulting from an estimation based on standardized output and respective input variables (zero mean, unit variance) to facilitate a comparison of their relative importance.

**Table 4:** Estimated linear effects.

	*Production function* ($\eta_{it}^{\left(y\right)}$)			*Effects on inefficiency* ($\eta_{it}^{\left(u\right)}$)		
Variables	qual & res	qual only	res only	qual & res	qual only	res only
	(2)	(8)	(9)	(2)	(8)	(9)
*const*	0.470***	0.490***	0.609***			
*D*^*ObsMor*^	−0.329***	−0.306***				0.319***
*D*^*ObsReadm*^	−0.186***	−0.180***				0.246***
log(*ObsMor* *)	0.397***	0.420***				−0.517***
*stand. coeff*.	*0.339*	*0.358*				
log(*ObsReadm* *)	0.282***	0.314***				−0.287***
*stand. coeff*.	*0.236*	*0.262*				
log(*Phys*)	0.056***		0.264***		−0.235***	
*stand. coeff*.	*0.063*		*0.318*			
log(*Nurse*)	0.015*		0.073***		0.129***	
*stand. coeff*.	*0.016*		*0.077*			
2008	−0.025**	−0.025**	−0.053***	0.064	0.061	−0.076
2010	−0.085***	−0.085***	−0.144***	0.235***	0.217***	−0.063
2012	−0.107***	−0.106***	−0.175***	0.253***	0.241***	0.031
2013	−0.056***	−0.052***	−0.116***	0.362***	0.352***	0.146**
*MSStrDis*				−0.829***	−0.743***	−0.136
*MedDepCon*				−0.228**	−0.148	−0.909***
*NumStrHospDis*				0.009**	0.008**	−0.003
log(*PatShaStroke*)				−0.252***	−0.200***	0.003
*SUnonCert*				−0.592***	−0.584***	−0.020
*SUCert*				-1.167***	-1.295***	−0.056
log(*GPsPerDis*)				−0.004	−0.019	0.129
*Spec*				−0.022	−0.001	0.156***
log(*HosBed*)				−0.555***	−0.482***	−0.036
*PrivHos*				0.310***	0.251***	0.090
*NonProfHos*				0.158***	0.133***	−0.107**
*Teach*				−0.069*	−0.040	−0.038
*UniHos*				0.175	0.137	0.455***
*ShaTRHOMB*				0.052	0.185	-1.144**
*ShaI*61				0.619***	0.639***	−0.099
*ShaI*64				−0.299***	−0.303***	−0.258***
*DiagCon*				0.394***	0.318***	0.429***
$\sigma_{v}^{2}$	0.142	0.148	0.141			
$\sigma_{u}^{2}$	2.301	1.982	0.439			
$\gamma =\sigma_{u}^{2}/\left(\sigma_{v}^{2}+\sigma_{u}^{2}\right)$	0.9419	0.9305	0.7569			
*TE* (in %)	73.0	72.3	68.2			
*DIC*	2581.7	2856.3	3817.2			
*WAIC*	2742.3	2994.9	3956.7			

Significance levels:

*** 1%;

** 5%;

* 10%;

based on 5522 observations

#### Production function

Across all model specifications, the estimated output elasticities of the (non-zero) input variables are positive and significant. The coefficients of the dummy variables identifying zero input observations are significantly negative. These estimates can be interpreted as the mean number of risk-adjusted stroke patients, i.e., 0.7 (= exp(−0.329)) or 0.8 (= exp(−0.186)), if the observed mortality or readmission is zero, respectively. The negative year effects relative to the base year 2006 decrease over time until 2012 and, thus, represent a trend towards quality improvements. This might be explained through medical technology progress, improved evidence-based guidelines for care provision or health policy changes. Standardized output elasticities of both resource variables (physician and nurse staff levels) are smaller than the elasticities of the quality indicators. The higher proportionality between the output term (risk-adjusted patient volume, based on the expected 30-day mortality) and the quality input terms (observed 30-day mortality and readmissions) might serve as an explanation here. The standardized effect of the physician staff level is stronger than that of nurse staff levels, since physicians are critical for care provision and outcomes by making the crucial diagnostic and treatment decisions. The output elasticity of observed mortality is about 44% higher than that of 30-day readmissions. Relative to readmissions, observed mortality has a higher proportionality with risk-adjusted patient volume, which is calculated based on each hospital´s expected mortality.

#### Effects on hospital performance

The estimated inefficiency effects for the different models offer some evidence for a trade-off between quality improvement and resource reduction. For instance, in Model 9, where quality is neglected in the production function, the negative effects of both quality indicators on the inefficiency term suggest that hospitals offering worse quality are able to provide stroke care relatively more resource efficient. Similarly, the effects of both dummy variables, *D*^*ObsMor*^ and *D*^*ObsReadm*^, identify high quality hospitals (no deaths or readmissions) as relatively less efficient. This result underlines the importance to expand the traditional approach of considering only costs or resources by integrating quality measures in hospital performance assessment. The estimated effect of physicians (log(*Phys*)) differs substantially from that of nurses (log(*Nurse*)) in Model 8, in which physicians (nurses) have a positive (negative) effect on quality performance. However, this interpretation might be misleading due to the high correlation of both input terms (> 0.94, unconditionally), which might explain the negative correlation of the coefficients. To clarify this issue, we have applied a Principal Component analysis to separate potentially different effects that are masked within both variables. We have found a negative effect on inefficiency of the component, which explains about 97% of the variation and highly correlates with both input variables (> 0.99). The second component, which correlates negatively (positively) with nurses (physicians), has also a negative effect. Although explaining less than 1% of the variation, the second component might govern the positive effect of the variable log(*Nurse*) on inefficiency.

The two variables measuring centralization have both negative effects on the inefficiency. A comparison of the results across the models offers a decomposition of the efficiency improvements, since the strength of the effects varies across the model specifications. The impact of market (*MSStrDis*_*it*_) and of the number of stroke care hospitals in one district (*NumStrHospDis*) is weaker when quality is not taken into account (Model 9). The concentration of stroke patients in medical departments (*MedDepCon*_*it*_) has a stronger effect in comparison with models including quality (Model 2 and 8). Thus, the internal concentration (of stroke patients within the hospital across medical departments) affects mostly the resource use, while the external concentration (market share and number of stroke care hospitals) has stronger effects on the quality component of efficiency.

Specialization in stroke treatment, relative to other conditions (*PatShaStroke*_*it*_), and (non-certified) stroke-units (*SUnonCert*_*it*_) have no effect on resource efficiency. However, if outcome quality of care is taken into account, both specialization measures have significantly negative effects on inefficiency. Similarly, the certification of stroke units (*CertSU*_*it*_) enhances quality performance (Model 2, Model 8), but not resource efficiency (Model 9). Accordingly to Model 2, hospitals with a DSG-certified stroke unit exhibit less inefficiencies than hospitals with non-certified stroke units (−1.167 vs. −0.592).

Similar to other literature on hospital performance, private, for-profit hospitals face higher inefficiencies than public hospitals [33, 90]. In our estimations, the latter result holds for private, non-profit hospitals only if quality is taken into account. The effect is negative if the performance is assessed only in terms of resources and contradicts with previous findings [33, 91]. The financial straits for private, non-profit hospitals [92] might have provoked cost containment efforts, by increasing resource efficiency at the cost of quality performance.

The respective performance differential for university hospitals varies over the input specifications. It is only significant if quality is excluded. On average, university hospitals treat more severe cases with a higher CMI compared with non-university acute care hospitals. Including quality measures corrects for the more complex and resource intensive tertiary care provided by university hospitals.

The shares of the different stroke types, *sh* − *I*61_*it*_ and *sh* − *I*64_*it*_, obtain effects which are in line with expectations. The treatment of intracerebral hemorrhage (I61) is more complex and faces higher risks and mortality for the patients in comparison with the treatment of the reference group, i.e., ischemic stroke (I63). Furthermore, with a higher share of I61, the critical diagnostic and therapeutic distinction between I61 and I63 needs to be undertaken more frequently, which increases complexities. However, these complexities do not affect resource efficiency. Patients with an early stage or preliminary stroke, not classified as hemorrhage or ischemic stroke, (I64) are treated most efficiently, irrespective of whether quality is accounted for or not.

In contrast to our expectation, a higher concentration of stroke type diagnoses (*DiagCon*_*it*_) enhances inefficiencies. Higher concentration on one particular diagnosis might result in diseconomies of scale. Hospitals that face a high concentration of relatively low resource consuming stroke patients (e.g., with diagnosis I64) might also hold ready fixed resources for eventually more complex stroke admissions. With regard to the latest treatment innovations, a higher number of thrombectomy procedures increases resource efficiency, possibly due to a shorter length of stay [93]. However, *ShaTRHOMB*_*it*_ has no significant effect in the combined quality performance and resource efficiency model, which might be explained by thrombectomy being used in more severe cases, with severity not directly captured in the QSR risk-adjustment.

Similar to the centralization of stroke treatment at the district level, hospital beds have a negative effect on inefficiency only if quality is accounted for. Larger hospitals with more beds might benefit from improved economies of scale and more experience resulting in higher quality. Resource efficiency is not affected by hospital size.

#### Spatial patterns of hospital performance

**Fig 1:**
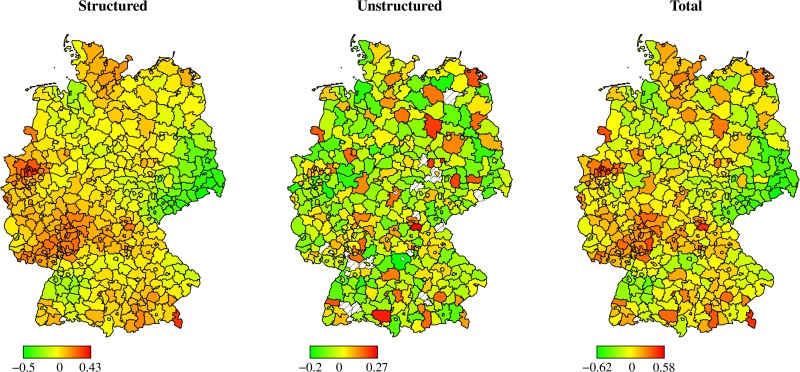
Centered estimated regional random effects of Model 2: Quality & resources.

To visualize the spatial pattern of the regional effects entering the predictor of the inefficiency term ($\eta_{it}^{\left(u\right)}$) in Eq (7), we display its centered posterior mean in Fig 1 for each German district. Non-colored, white areas indicate districts without sample observations. In contrast to the structural spatial effect (left hand side), the unstructured spatial effect (center) contributes to the composite spatial effect (right hand side) with markedly less variation. In total, favourable local conditions for the treatment of stroke patients are detected, in particular, for hospitals in Eastern and Southern Germany, while hospitals in North-Eastern and Western Germany are characterised by higher inefficiencies in the treatment of stroke patients.

**Fig 2:**
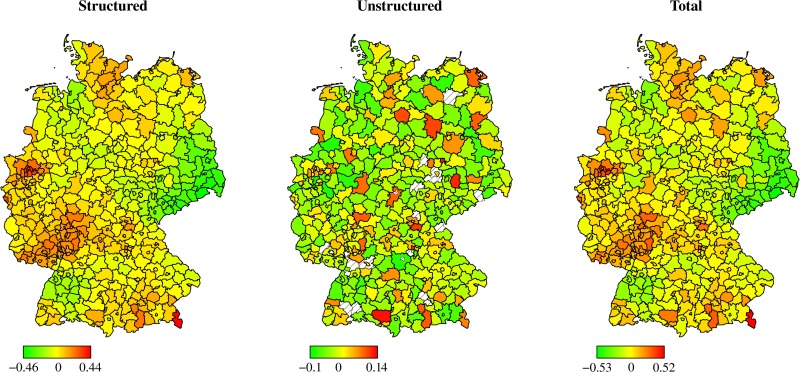
Centered estimated regional random effects of Model 8: Quality only.

**Fig 3:**
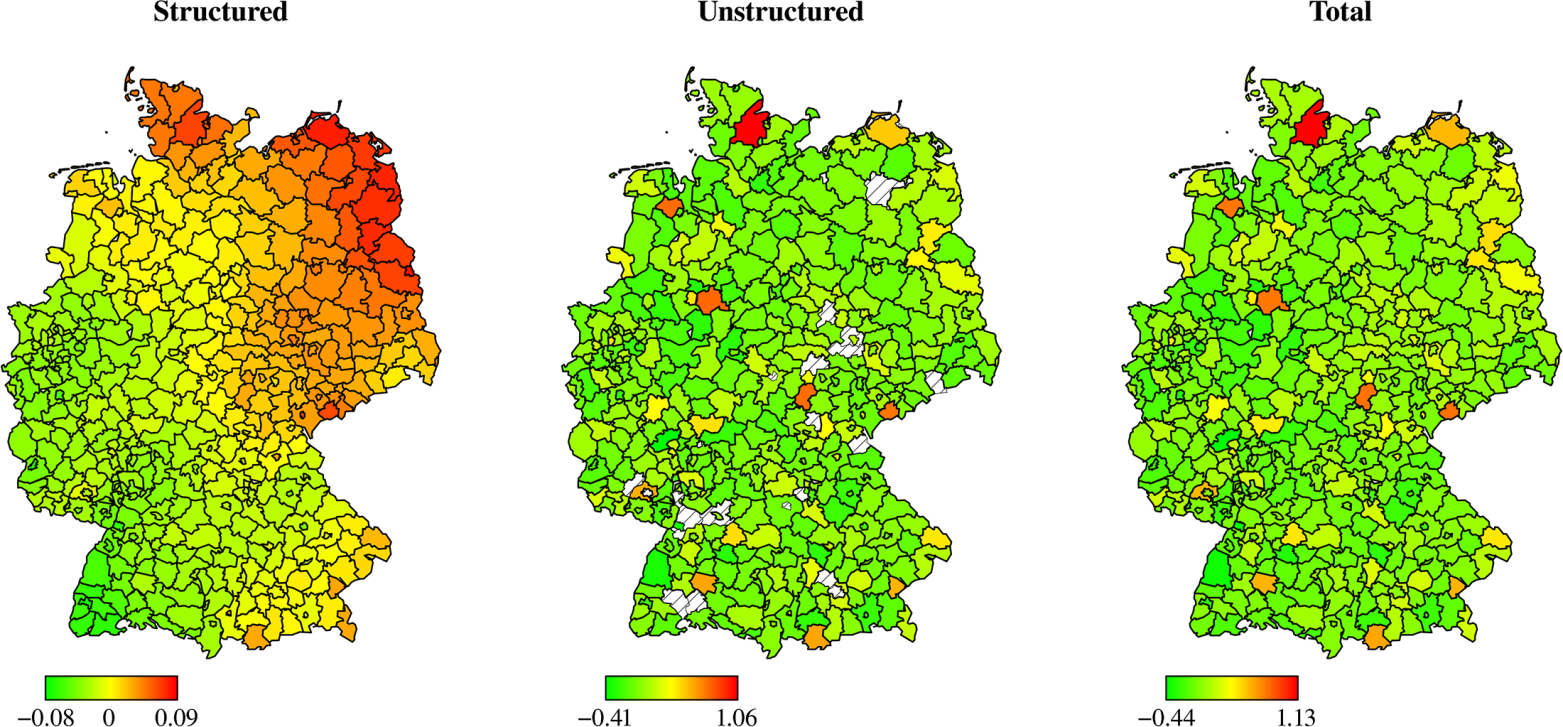
Centered estimated regional random effects of Model 9: Resources only.

A spatial decomposition of the inefficiencies into quality (deceased patients, Fig 2) and resources (staff levels, Fig 3) underlines the previous finding that an efficient use of resources is not always in line with high quality treatment of patients. While some regions in Western Germany show an efficient use of resources (e.g., within North Rhine-Westphalia or Rhineland-Palatinate), they lack behind other regions in Eastern Germany (e.g., within Brandenburg or Saxony) if quality performance is considered. However, some regions (e.g., within Baden-Wuerttemberg) perform well in terms of quality performance as well as resource efficiency.

### 3.3 Estimated (in)efficiencies

#### Slack resources

**Fig 4:**
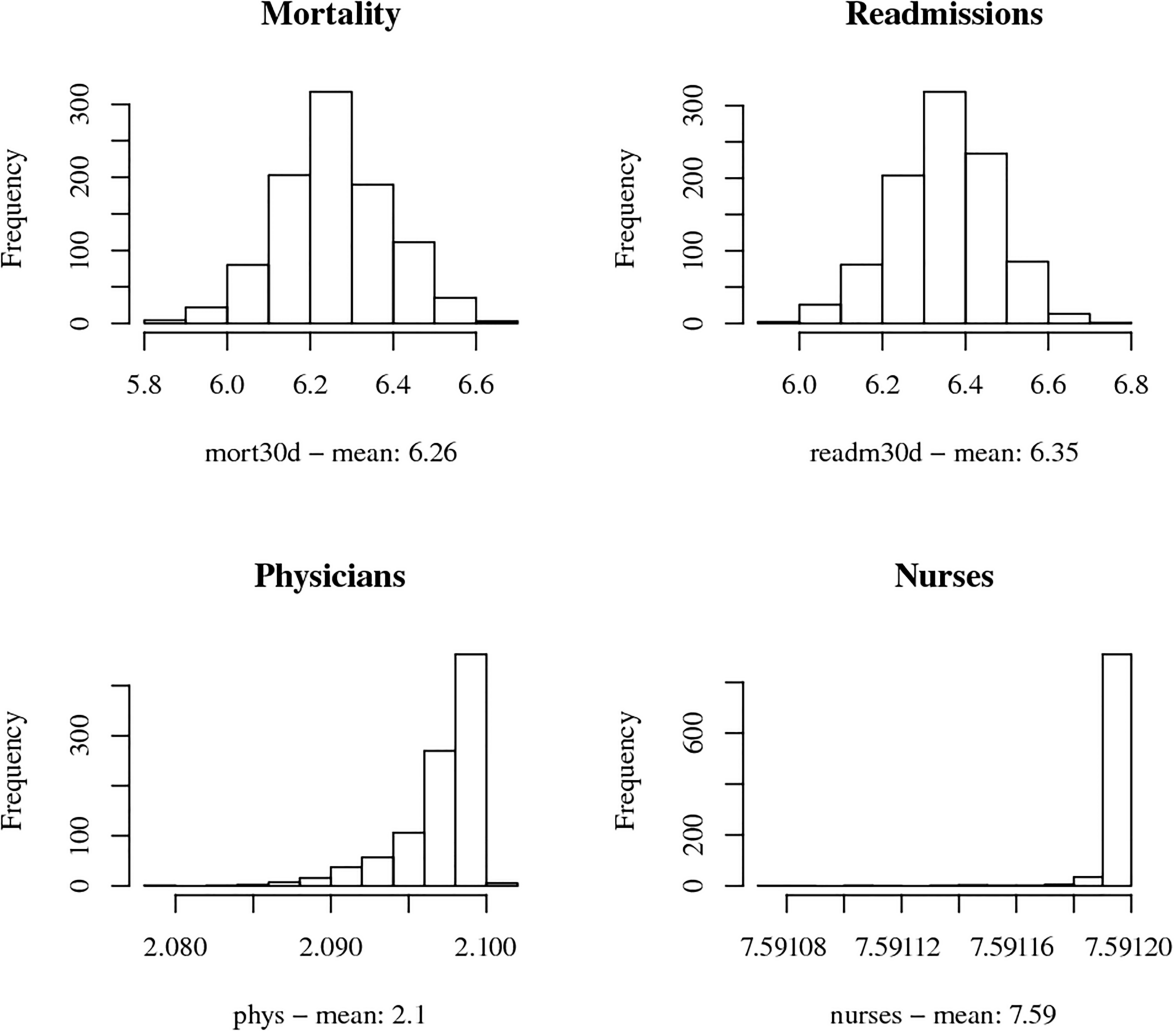
Posterior distribution of slack resources.

Accordingly to the best fitting quality-and-resource Model 2, the overall posterior mean of technical efficiency is 0.730, indicating a sizeable potential for quality improvement and/or resource reallocation. To quantify such potentials for an average hospital (sample means of all input and explanatory variables), we compute slack resources, i.e., resources in excess of those needed under full efficiency (for methodological details see eq (12) in S1 Appendix). We show the respective posterior distributions in Fig 4 for each input variable (in levels). If the average hospital boosts its performance to an efficient treatment of stroke patients, the mortality can be reduced by up to 6.26 (63.70%) deaths per year, which is close to the maximum quality improvement for readmissions. In 2013 the means of observed mortality and readmission were 9.83 and 8.82, respectively. Similarly, we can also compute the slack resources for each observation evaluated at the (MCMC) posterior mean, obtaining for 2013 total numbers of nation-wide saved deaths and readmissions of 2630 (24.62%) and 2951 (30.78%), respectively.

#### Marginal effects

To highlight the economic relevance of those variables that describe the inefficiency, we provide their marginal effects on both technical efficiency and slack resources. If the latter are positive or negative, the effect quantifies how much of an input can be reduced or increased to achieve the same level of output (for methodological details see eqs (9) and (13) in S1 Appendix). All effects shown in Table 5 are evaluated at the means of the underlying variables. Of particular interest are the marginal effects of treating patients in certified and non-certified stroke units, concentrating stroke treatment in fewer medical departments at the hospital level, and concentrating stroke treatment in fewer hospitals at the district level.

**Table 5:** Marginal effects on efficiency and slack resources (Model 2).

Variables	Marginal effect on *TE* (×100)	Marginal effect on slack resources			
		*mort*30*d*	*readm*30*d*	*phys*	*nurses*
*Spec*	0.027	−0.010	−0.012	−0.016	−0.204
*MSStrDis*	1.253***	−0.453***	−0.533***	−0.566***	−5.361*
*NumStrHospDis*	−0.015**	0.005**	0.006**	0.008**	0.110
log(*PatShaStroke*)	0.400***	−0.148***	−0.175***	−0.202***	−2.531*
log(*HosBed*)	0.888***	−0.324***	−0.382***	−0.420***	−4.455*
*PrivHos*	−0.449***	0.170***	0.202***	0.255***	4.267*
*NonProfHos*	−0.258***	0.097***	0.115***	0.145***	2.227*
*Teach*	0.092	−0.034	−0.041	−0.049	−0.664
*UniHos*	−0.235	0.089	0.106	0.143	2.157
*MedDepCon*	0.319**	−0.118**	−0.140**	−0.166**	−2.058
*ShaTRHOMB*	−0.016	0.011	0.016	−0.004	0.161
*ShaI*61	−1.000***	0.384***	0.460***	0.624***	13.159*
*ShaI*64	0.482***	−0.178***	−0.210***	−0.241***	−2.862*
*DiagCon*	−0.627***	0.239***	0.285***	0.368***	6.696*
*SUCert*	2.660***	−0.927***	−1.079***	−1.012***	−7.016*
*SUnonCert*	1.105***	−0.401***	−0.472***	−0.508***	−5.027*
log(*GPsPerDis*)	0.108	−0.040	−0.047	−0.064	−0.841
2008	−0.087	0.033	0.039	0.047	0.668
2010	−0.314***	0.118***	0.141***	0.174***	2.779*
2012	−0.357***	0.135***	0.160***	0.200***	3.294*
2013	−0.499***	0.189***	0.226***	0.288***	5.014*

Significance levels:

*** 1%;

** 5%;

* 10%

For example, hospitals with a DSG-certified stroke unit are characterized by a 0.026 (3.3%) higher technical efficiency in comparison with hospitals that treat stroke patients but do not operate a stroke unit. By closing their lacks of efficiency, these hospitals could increase their treatment quality by reducing stroke mortality by 0.927 or readmissions by 1.079 per year. When moving stroke treatment from hospitals without a stroke unit to hospitals with a non-certified stroke unit, mortality (readmissions) can be reduced by 0.401 (0.472).

### 3.4 Robustness checks

To validate our results, we subject our analysis to a series of robustness checks, based on our best fit quality-and-resource Model 2. Results are presented in Table 6 for key variables.

**Table 6:** Robustness-checks.

selected Variables	base-line model	Translog prod-fun	rescaling zero-inputs	*SUnonCert*		*SUCert* age	health variables	*f*_*str*_(*dist*_*i*_) & *f*_*unstr*_(*NUTS2*_*i*_)	*f*_*str*_(*NUTS2*_*i*_) & *f*_*unstr*_(*dist*_*i*_)	matching & DID
				(> 5)	(> 20)					
	(2)	(10)	(11)	(12)	(13)	(14)	(15)	(16)	(17)	(18)
*MSStrDis*	−0.829***	−0.892***	−0.843***	−0.816***	−0.803***	−0.812***	−0.799***	−0.845***	−0.839***	
*MedDepCon*	−0.228**	−0.228**	−0.212**	−0.213**	−0.211**	−0.202**	−0.205**	−0.180**	−0.216**	
*NumStrHospDis*	0.009**	0.009*	0.008*	0.009**	0.009**	0.009**	0.008*	0.004	0.011**	
log(*PatShaStroke*)	−0.252***	−0.207***	−0.245***	−0.258***	−0.256***	−0.257***	−0.258***	−0.253***	−0.262***	
*SUnonCert* (> 10)	−0.592***	−0.669***	−0.603***			−0.587***	−0.587***	−0.567***	−0.592***	
*SUnonCert* (> 5)				−0.489***						
*SUnonCert* (> 20)					−0.665***					
*D*^*m*^ × *SUnonCert* (> 10)										−1.370***
*SUCert*	−1.167***	−1.265***	−1.182***	−1.098***	−1.197***		−1.163***	−1.108***	−1.194***	
*SUCert* (1-2y)						−1.252***				
*SUCert* (3-4y)						−1.070***				
*D*^*m*^ × *SUCert* (DID)										−0.580**
(1 − *D*^*m*^) × *SUCert*										−2.259***
log(*GPsPerDis*)	−0.004	−0.007	−0.040	−0.058	−0.072	−0.060	−0.037	−0.135	−0.125	
log(*MortalityDis*)							−0.069			
log(*PopageDis*)							−0.065			
log(*UnemplrateDis*)							0.087			
log(*GDPpCapDis*)							0.010			

Significance levels:

*** 1%;

** 5%;

* 10%;

*D^m^* is a dummy variable: 1 for observations of matched hospitals and 0 else

As a more general formulation of our Cobb-Douglas production function, we estimate a translog model (10) and obtain very similar results for all relevant coefficients and model fit diagnostics. Likewise, rescaling the quality measures by adding 0.5 to each observation prior to log-transformation (Model 11) to handle optimal realized quality (zero mortality and/or readmissions) inputs achieves identical results as our first choice dummy variable approach.

To test alternative specifications of the non-certified, stroke unit variable *SUnonCert*_*it*_, we half and double the required number of complex stroke procedures for stroke unit identification, based on our baseline of 10 procedures. As before, the results of these models (12-13) are qualitatively equivalent. Additionally, we add in Model 14 variables that differentiate between younger (1-2 years) and older certificates (3-4 years). The results are comparable, but older certificates show a weaker effect (−1.070) than younger certificates (−1.252). This is possibly due to the fact that organizational processes and structural provisions optimized before and during the certification process might loose some rigour over time.

The robustness of the specified spatial structure is tested by including in Model 15 several district-level control variables such as annual mortality per 1,000 inhabitants, the average age of the population and unemployment rate in addition to the spatial structures. These variables do not improve model accuracy and their estimated effects are insignificant. This reflects the inclusion of regional health status due to the risk-adjusted patient population of each hospital and the structured and unstructured spatial structures. In addition, we examine two mixed-level models with NUTS2-level structured effects and district-level unstructured effects and vice versa (Models 16-17). Results are identical to our base-line Model 2.

Lastly, we safeguard the results of our certification variable for causal inference. Due to a potential self-selection of better performing hospitals pursuing certification, the existence of reverse causality cannot be excluded (see e.g. the findings of [94]). We adapt the procedure of [95] to the SFA context and apply in Model 18 a combination of a matching approach and a Difference-In-Difference (DID) estimation to investigate the impact of certification on hospital performance. In a first step, we use propensity-score matching to ensure that any observed differences between certified and non-certified hospitals can be attributed to certification. To achieve a balance between both groups of hospitals in their baseline characteristics, we match each hospital becoming certified during the sample period with a non-certified hospital. In a second step, we estimate a DID specification of our best fitting Model 2. We include all observations in the estimation to achieve a comparable production frontier. However, we model specific effects on the inefficiency term for the pairs of matched hospitals. We also estimate specific effects for non-matched hospitals. The results confirm the previous finding that hospital inefficiency is reduced by certification; however, the effect in the DID Model 18 is smaller.

## 4 Discussion

In the following, we highlight four main implications. First, we discuss the improved efficiency for certified stroke units and the potential quality benefits of treating stroke patients in stroke units only, as well as the possible gains from within-hospital concentration of stroke care. Second, we examine the potential for resource reallocations due to efficiency improvements associated with specialization, certification and concentration. Third, we stress the potential benefits from regional concentration of stroke care. Fourth, we comment on the regional differences in hospital technical inefficiencies. In addition, we address some shortcomings of dataset and methodology.

### Stroke unit specialization and certification

In general, health policy makers, regulators, provider groups and payers could initiate substantial further consolidation and concentration of stroke care in certified centers of excellence. In 2013, 136,000 stroke patients (49%) were treated in certified stroke units while 80,000 patients (29%) were treated in non-certified stroke units and 60,000 (22%) stroke patients were treated in hospitals without a stroke unit (see Table 1). Certified and non-certified stroke units, on average, have a risk-adjusted stroke mortality of 0.9, while hospitals without a stroke unit have a risk-adjusted mortality of 1.0. A large share of stroke patients has not been treated in the best manner. Our results highlight the benefit of (further) care specialization and certification.

The twice-as-large efficiency gain for hospitals with certified stroke units can be explained by the increased expertise, better infrastructure and higher service level requirements for DSG-certified stroke units. For example, the DSG requires in its stroke unit manual a minimum of 250 annual stroke patients treated and a minimum level of 1.5 full time equivalent specialized nursing staff per stroke unit bed for a local stroke unit [96]. In preparation for the DSG audits, the stroke care team reviews and updates process plans, which can lead to improved outcome quality and efficiency after the certificate is granted. As indicated by the certification timing effect identified in the robustness section confirms, the effect is particular strong in 1-2 year after certification.

Exploiting the quality differences between the different stroke treating hospitals at the national level can result in substantial quality of care improvements and reductions of annual stroke deaths. If all stroke patients that are currently treated at hospitals without a stroke unit were treated at hospitals with a non-certified stroke unit, the average 30-day mortality for the 937 hospitals without a stroke unit could be reduced by 0.401 deaths, ceteris paribus. At the country level, this could result in annually 376 fewer stroke deaths after 30 days (i.e., ≈1% decrease of German national stroke mortality in 2013). Even more, if all patients from hospitals without a stroke unit were treated at hospitals with a certified stroke unit, national stroke mortality could be reduced by 868 deaths (≈2% reduction), ceteris paribus. Similar benefits could be achieved for readmissions. With regard to optimal resource allocation, not all currently stroke-treating hospitals shall set-up a (certified) stroke unit, but instead, centralizing stroke care in those hospitals that run a high-quality (i.e., certified) stroke unit could result in a more efficient and higher quality stroke care provision. To implement this concentration, a two stage policy approach appears feasible. In the first stage, regulators might require that stroke patients can only be treated in stroke units and, in a second stage, in DSG-certified stroke units only.

### Intra-hospital concentration

Next to specialization and certification, hospitals can also concentrate their acute and rehabilitative care within a specialized medical department or stroke unit. Depending on size and specialization, hospitals often undertake care for one treatment area in several medical departments. Stroke care is a particularly good example as stroke diagnostic and therapeutic interventions can be performed by, e.g., the internal medicine, cardiology, and neurology departments. In 2013, only 381 hospitals provided stroke care in one medical department, 261 hospitals treated stroke patients in two medical departments, 128 hospitals treated stroke patients in 3 medical departments, and 339 hospitals treated stroke patients in at least 4 medical departments.

The negative and significant coefficient for hospital-level stroke care centralization (*MedDepCon*_*it*_) in Model 2 (see Table 4) indicates that, in fact, organizational care and process changes to enhance within-hospital centralization can improve efficiency and care outcome quality. Following acute treatment in a stroke unit, patients can continue to be treated on this same specialized stroke unit or within a less intensive care setting rather than being relocated to different wards based on free hospital bed capacity. Considering the marginal effect for observed 30-day mortality of -0.142 and the average level of within-hospital, stroke care centralization of 0.83, mortality can be reduced by 0.023 deaths for the average hospital, if within-hospital stroke care centralization is maximally concentrated (at a HHI of 1). Extrapolating within-hospital effects to the national level, this could have a mortality reduction effect of 33 deaths per year.

### Regional concentration of stroke treatment

Regional concentration provides further potential for efficiency and outcome quality improvements. Currently, stroke care is undertaken by 1,391 hospitals (in 2013) in 415 districts in Germany. Case volumes range from 10 or less in the 110 hospitals with lowest volumes to 1,000 or more in the largest 27 stroke hospitals. There are almost 800 hospitals that provide care for less than 250 patients annually (DSG requirement for regional stroke unit), and 587 hospitals that provide care for less than 100 stroke patients. Likewise, in 2013 a single district hosts, on average, 7.5 hospitals that provide stroke care. There are 154 districts with 1 or 2 stroke hospitals, 173 districts with 3-5 hospitals, 60 districts with 6–10 hospitals, and 16 districts with more than 10 stroke hospitals.

To demonstrate the benefits from regional concentration, we highlight the inefficiency reducing effects of district hospital market share for stroke care. With a marginal effect of 0.013 on technical efficiency and an efficiency increasing elasticity of -0.453 for the average hospital, increases of the stroke hospital market share can substantially enhance efficiency and quality of care. When increasing the market share for half of the hospitals (696 hospitals) from an average of 30% to a share of 63% (mean plus one SD), annual national stroke mortality and readmissions could be reduced by 347 deaths and 408 cases per year, respectively, ceterius paribus.

Especially for emergency conditions, elapsed time until treatment is critical. In many countries, the recommended time window for stroke treatment after the onset of symptoms is 3.0 to 4.5 hours [97]. In their review of stroke treatment guidelines and studies, [97] emphasize the benefits of bypassing hospitals without a stroke unit in favor of treatment at more distant hospitals with a stroke unit. Similarly, several studies have shown the benefits of treating stroke patients in a more centralized model with specialized hospitals as opposed to a decentralized model with several smaller, non-specialized community hospitals [98, 99].

### Regional variation in technical efficiency

Focusing on resource efficiency only, some studies have found higher efficiencies for regions in Western and Northern Germany [100]. As a methodological distinction, however, we also include observed quality as an input and risk-adjust the output patient volume for specific patient risk-factors rather than considering CMIs. Furthermore, we evaluate efficiency for a specific treatment area and not for the overall hospital, which avoids the grouping of treatment areas. These methodological advances can possibly explain the following differences in the results. Including quality in the efficiency estimations, we find sizable inefficiencies for hospitals located in North Rhine-Westphalia (NRW), and, in particular, in the Rhein-Ruhr region. When excluding quality from the production function, hospitals located in NRW become relatively more efficient, which is in line with results in [100]. Interestingly, several regions (e.g., in Eastern Germany) with resource inefficiencies show a higher ability to produce better outcome quality.

### Trade-off between quality and resource inputs

Our results illustrate the managerial potential to reduce or reallocate resource inputs while keeping stroke care quality measured in terms of observed mortality and readmissions constant. As the marginal effects on the staff slack resources (see Table 5) demonstrate, the efficiency enhancing effects of specialization, certification, regional concentration, and higher stroke patient share are substantial with regard to staff resources. When specialization and concentration increase, the productivity of both nurses and physicians increases, which can free up resources for reallocation, or as described above, might be invested in quality improvements. While a difficult choice, clinical and administrative hospital managers have to regularly decide on how to allocate limited resources more efficiently. Modelling such trade-offs can support health services managerial resource allocation. Ideally, organizational inefficiencies can be reduced through a combination of quality of care improvements and resource reallocations. This will achieve the highest reduction of the inefficiency gap since the marginal effect from reallocated units decreases the closer one gets to the efficiency frontier. Managerial and policy interventions should address both dimensions simultanously.

### Limitations

With regards to the data and methodology employed, we consider several limitations, which might impact the interpretation of the results. The QSR outcome quality indicators are based on AOK patient-level data and address the quality of care for AOK stroke patients treated in each hospital. Hence, outcome quality data is limited to AOK insured patients and quality data for patients insured with other public sickness funds and private health insurers is not included. However, the AOK is by far the largest health insurer in Germany, with an overall market share of 35% among publicly insured patients and a range of state-level market shares between 21% and 51%. This indicates that AOK patient data covers a large share of inpatient treatments and lets us assume representativeness of the AOK outcome data [101]. Furthermore, the morbidity-oriented risk structure compensation scheme within the public sickness fund system indicates that AOK patients, on average, are older and have a weaker health status compared with the rest of the statutory health insurance (SHI) population [102]. While this could possibly lead to an overestimation of the potential inefficiencies, the majority of such a bias is controlled for when using risk-adjusted outcomes.

In addition, risk-adjustment based on administrative data, as opposed to clinical data, has limitations with regards to risk factors included. Most importantly, administrative data currently does not allow adjustments for disease severity levels and the degree of consciousness at admittance. Yet, the risk factors that are included in the risk-adjustment methodology, i.e. age and obesity, are shown to correlate with severity and consciousness. More general, risk-adjustment only accounts for measurable and reported risk-factors, with many important risk factors for adverse outcomes not measurable based on current methods of administrative data-based risk-adjustment, e.g., preoperative function status, or not consistently reported (e.g., obesity) [103].

Furthermore, mortality and readmissions are only two, albeit important, aspects of stroke quality of care. Other factors such as health-related quality of life [104] and patient-reported outcomes such as pain, selfcare, mobility, and health gain [105], are also important, and can have even stronger impacts on hospital choice [106]. As our results demonstrate, including multiple outcome quality aspects is important. When we include 30-day readmission only as an inefficiency determinant, an increase in 30-day readmissions reduces inefficiencies and indicates a trade-off between mortality and readmission as two distinct components of outcome quality [107]. Lastly, the laboratory used for this study was the German hospital landscape and results on other European countries might differ. A cross-country analysis at the hospital-level is in general not possible due to limited data availability and comparability of hospital level quality and structural data across countries.

## 5 Conclusions

In order to estimate a quality-expanded notion of hospital efficiency, we employ an innovative geoadditive SFA model with realized quality as an input and risk-adjusted patient volume as output. Past hospital efficiency research has mostly neglected quality of care, due to the difficulties in and drawbacks of risk-adjustment techniques and data availability. Research has also often neglected the potential for spatial patterns of inefficiency, which is especially problematic in local and regional settings with specific legislation and demand patterns such as hospital markets.

To address these shortcomings, we expand the notion of technical efficiency in three important directions. First, we include measures of realized poor quality as input variables for the production function. Second, we develop a standardized and risk-adjusted patient volume output measure based on the standardized mortality ratio. Third, we include spatial factors as inefficiency determinants. Altogether, these three methodological advances improve the applicability of technical efficiency estimations in a hospital service provision setting and can possibly increase the relevance and adoption of efficiency modelling in health policy making. This expanded notion of technical efficiency also informs efficiency estimations in broad service operations research.

Our findings confirm that quality of care as well as spatial structures are highly important in shaping hospital technical efficiency. An overall hospital efficiency mean of 0.730 highlights the efficiency losses for the average hospitals, with regard to both quality and resource usage in stroke care. These inefficiencies have several important determinants. Specialization through a stroke unit improves outcome quality of care and resource efficiency. These effects are further strengthened if the stroke units are certified in accordance with highest standards (DSG certification). Within-hospital stroke care concentration at one medical department as well as regional stroke care concentration can also reduce inefficiencies and annual stroke mortality. Based on the marginal effects on slack resources, we highlight substantial quality of care improvement potentials at both the hospital and the national level.

With regards to data and methodology used, some drawbacks exist. Using outcome quality indicators based on AOK patient data only, using only two outcome quality criteria and the underlying risk-adjustment methodology not including severity and consciousness at admittance are important limitations. However, the impact of the limitations is sufficiently small to ensure validity, reliability and generalizability of the results.

In general, this work provides clear evidence for the importance of including quality of care in hospital and other service operations efficiency modelling. The examined setting—a regulated market with a mix of private for-profit, private non-profit and public hospital ownership, with fixed prices and in the short-to-mid term fixed capacities—is common in many health care systems. Operationalizing the increasingly important and widespread concept of risk-adjusted, hospital-level mortality in a quality-enhanced SFA production function enables more comprehensive technical efficiency estimates in all countries, with available measures of standardized mortality ratios at a medical condition level (e.g., Germany, the US, the UK, and the Netherlands). Government agencies, health care regulators, and larger hospital chains (e.g., NHS Care Quality Commission in England, or the Hospital Corporation of America) can adopt the methodology to assess a quality-expanded notion of technical efficiency for their respective markets and hospitals. At a medical condition level, best practice hospitals providing optimal quality of care given their patient, resource and regional market constraints can be identified. Furthermore, medical care can be concentrated at these centers of excellence to increase both the quality of care provided to patients, and optimize resource utilization in health service provision. Additional research can expand the methodology to other treatment areas, such as other emergency conditions like acute myocardinal infarction or elective procedures such as hip and knee replacement.

## Supporting information

S1 Appendix Marginal effects. (PDF)
