# Supplementary material for: Quality and resource efficiency in hospital service provision: A geoadditive stochastic frontier analysis of stroke quality of care in Germany
Source: PLoS One. 2018 Sep 6;13(9):e0203017. doi: 10.1371/journal.pone.0203017 (PMC6126832; doi:10.1371/journal.pone.0203017)
Supplement: S1 Appendix — (PDF) [file pone.0203017.s001.pdf]

Marginal effects

In this section we, firstly, provide the formulas to obtain the marginal effects of the explanatory variables describing the inefficiency u on the estimated technical efficiency. Secondly, the determination of slack resources and the marginal effects on the slack resources are derived.

On technical efficiency

For notational convenience we suppress the indices t and i. The technical efficiency (TE) of the i-th hospital at time t is then obtained as [1]

$$TE = \mathrm{E}[\exp(-u|\epsilon)] = \frac{\exp\left(-\tilde{\mu} + \frac{1}{2}\tilde{\sigma}^2\right) \Phi\left(\frac{\tilde{\mu}}{\tilde{\sigma}} - \tilde{\sigma}\right)}{\Phi\left(\frac{\tilde{\mu}}{\tilde{\sigma}}\right)} = a \cdot b \cdot c, \tag{8}$$

where

$$\begin{aligned}
a &= \exp\left(-\tilde{\mu} + \frac{1}{2}\tilde{\sigma}^2\right) \\
b &= \Phi\left(\frac{\tilde{\mu}}{\tilde{\sigma}} - \tilde{\sigma}\right) \\
c &= \left[\Phi\left(\frac{\tilde{\mu}}{\tilde{\sigma}}\right)\right]^{-1} \\
\tilde{\mu} &= \frac{-\epsilon\sigma_u^2}{\sigma_u^2 + \sigma_v^2} \\
\tilde{\sigma}^2 &= \frac{\sigma_u^2\sigma_v^2}{\sigma_u^2 + \sigma_v^2} \\
\sigma_u^2 &= \left(\sigma_u^*\right)^2 \alpha = \left(\sigma_u^*\right)^2 \exp\left(\eta^{(u)}\right) = \left(\sigma_u^*\right)^2 \exp\left(z'\beta^{(u)}\right) \\
\epsilon &= y - \eta^{(y)}.
\end{aligned}$$

The marginal effect (ME_k) of the k-th explanatory variable on TE is given by

$$\begin{aligned}
ME_k = \frac{\partial TE}{\partial z_k} &= a\left(\frac{1}{2}d - e\right) \cdot b \cdot c \\
&\quad + a \cdot \phi\left(\frac{\tilde{\mu}}{\tilde{\sigma}} - \tilde{\sigma}\right)(g - f) \cdot c \\
&\quad - a \cdot b \cdot c^2 \cdot \phi\left(\frac{\tilde{\mu}}{\tilde{\sigma}}\right) g,
\end{aligned} \tag{9}$$

respectively, where

$$
\begin{aligned}
d &= \beta_k^{(u)} \frac{\tilde{\sigma}^2}{\sigma_u^2} \\
e &= -\epsilon \beta_k^{(u)} \frac{\sigma_u^2 \sigma_v^2}{\left(\sigma_u^2+\sigma_v^2\right)^2} \\
f &= \frac{1}{2} \sigma_u \sigma_v \beta_k^{(u)} \left(\left(\sigma_u^2+\sigma_v^2\right)^{-\frac{1}{2}} - \left(\sigma_u^2+\sigma_v^2\right)^{-\frac{3}{2}} \sigma_u^2 \right) \\
g &= \frac{\tilde{\sigma} e - \tilde{\mu} f}{\tilde{\sigma}^2}.
\end{aligned}
$$

On slack resources

To quantify the potential input reduction for a given (risk-adjusted) output level due to improvement of efficiency, we start by considering a simple SFA model in levels

$$
\mathrm{E}[Y] = \prod_{k=1}^{K} X_k^{\beta_k} \cdot TE,
$$

where $TE = \mathrm{E}[\exp(-u|\epsilon)] \in (0,1)$ (see (8)) denotes the technical efficiency. An increase of TE offers a reduction of the inputs if the output level is not changed. Formally, an increase of TE by ΔTE relates to a reduction of the deterministic part of the production function, $\prod_{k=1}^{K} X_k^{\beta_k}$, by $\frac{TE}{TE+\Delta TE}$, since

$$
\mathrm{E}[Y] = \prod_{k=1}^{K} X_k^{\beta_k} \cdot TE = \prod_{k=1}^{K} X_k^{\beta_k} \frac{TE}{TE+\Delta TE} \cdot (TE+\Delta TE).
$$

Defining the reduced part involving the input variable of interest, say X_k, as

$$
(X_k^*)^{\beta_k} = X_k^{\beta_k} \frac{TE}{TE+\Delta TE} = \frac{X_k^{\beta_k}}{1+\frac{\Delta TE}{TE}}
$$

and solving it for the new level of input X^*

$$
X^* = \frac{X_k}{\left(1+\frac{\Delta TE}{TE}\right)^{1/\beta_k}},
$$

the reduction of X_k due to an increase of TE by ΔTE is given by

$$
\Delta X_k = X_k^* - X_k = X_k \left(\frac{1}{\left(1+\frac{\Delta TE}{TE}\right)^{1/\beta_k}} - 1 \right).
$$

If for ΔTE the difference of being efficient, $\Delta TE = 1 - TE$, is considered, the slack resources are determined by

$$
slack_k = X_k - X_k^* = X_k \left(1 - TE^{1/\beta_k}\right). \tag{10}
$$

By inserting for ΔTE the marginal effect of an explanatory variable describing the inefficiency u on the technical efficiency TE, $\Delta TE = ME$, we quantify the marginal effect of z on the slack resources as

$$
\Delta X_k = X_k^* - X_k = X_k \left(\frac{1}{\left(1+\frac{ME}{TE}\right)^{1/\beta_k}} - 1 \right). \tag{11}
$$

References

1. Jondrow J, Lovell CAK, Materov I, Schmidt P. On the estimation of technical inefficiency in the stochastic frontier production function model. Journal of Econometrics. 1982;19:233–238.
